# Whole genome sequences of highly pathogenic H5N1 avian influenza viruses detected in felines in Bangladesh

**DOI:** 10.1128/mra.00835-26

**Published:** 2026-10-16

**Authors:** Sajedul Hayat, Shukes Chandra Badhy, Mohammad Enayet Hossain, Ashis Kumar Kundu, Salima Ferdows, Safeth Arju, U. K. Fatima Zannat, Tasnim Sunjana, Yamin Ali, Mohammad Sadekuzzaman, Md. Golam Azam Chowdhury

**Affiliations:** 1 Central Disease Investigation Laboratory, Department of Livestock Services, Ministry of Fisheries and Livestock, Dhaka, Bangladesh; 2 International Center for Diarrhoeal Disease Research, Bangladesh (icddr’b) 56291, Dhaka, Bangladesh; 3 Department of Biomedical Engineering, Khulna University of Engineering & Technology 130060, Khulna, Bangladesh; DOE Joint Genome Institute, Berkeley, California, USA

**Keywords:** H5N1, clade 2.3.4.4b, Bangladesh, mammalian adaptation, avian influenza virus

## Abstract

Whole-genome sequences of three highly pathogenic H5N1 avian influenza viruses detected in cats in Bangladesh during 2025–2026 are reported. Phylogenetically, two strains grouped within clade 2.3.2.1a, and the other within the clade 2.3.4.4b. The isolates harbored mutations in key polymerase and HA markers associated with mammalian adaptation and virulence.

## ANNOUNCEMENT

Highly pathogenic avian influenza (HPAI) A (H5N1) belongs to the genus *Alphainfluenzavirus* of the *Orthomyxoviridae* family (1) and is a severe, often fatal viral infection that can cross species barriers to cause severe respiratory and neurological disease in mammals, including Felidae (2, 3). In April 2025, the first HPAI case in two imported serval cats (*Leptailurus serval*) was reported in Bangladesh (4), and three other cases were reported in domestic cats (*Felis catus*) brought to the Central Disease Investigation Laboratory (CDIL) for diagnosis (Table 1). In this announcement, we present the coding-complete genome sequence of three H5N1 strains isolated from three of the four cases.

**TABLE 1:** Sample provenance, assembly information, and FluSurver strain identification and mutation profiling of the sequenced Bangladeshi H5N1 strains submitted by the Central Disease Investigation Laboratory, Bangladesh

Isolate ID	CDIL_AIV_H5N1_1388	CDIL_AIV_H5N1_2307	CDIL_AIV_H5N1_2675
Host/location	Serval cat, Narayanganj	Domestic cat, Dhaka	Domestic cat, Dhaka
Case History	6 months of age, rapid death with high fever (107°F–108°F).	10 months of age, death; history of ovariohysterectomy, followed by postoperative infection	2 years of age, suspected cat flu
Sample	Tracheal swab	Tracheal swab	Oropharyngeal swab
Collection date	April 20, 2025	October 30, 2025	January 22, 2026
Sequenced read and GC%	7,074, 43.2%	3,222, 45.0%	4,369, 44.9%
Read length N50 (bp)	559	787	512
Mean Q (fastq_pass, Q ≥ 9)	12.2	12.3	12.4
Mean coverage (For HA, NA, PB2, PB1, PA, NP, MP, and NS)	189.75x, 139.69x, 28.42x, 119.01x, 77.77x, 58.79x, 343.95x, and 646.36x	76.54x, 97.12x, 19.72x, 59.91x, 86.21x, 71.94x, 223.53x, and 507.59x	26.84x, 29.02x, 189.50x, 221.88x, 78.15x, 27.28x, 169.57x, and 280.53x
Segments sequenced	8 segments	8 segments	8 segments
Genome size	13,528 bp	13,528 bp	13,430 bp
HA clade	2.3.2.1a	2.3.2.1a	2.3.4.4b
GenBank assembly	GCA_058941575.1	GCA_058941755.1	GCA_058941855.1
HA ref strain, % AA Identity	A/Hubei/1/2010, 95.6%	A/Hubei/1/2010, 95.4%	A/Astrakhan/3212/2020, 98.2%
NA ref strain, % AA Identity	A/Hubei/1/2010(H5N1), 93.98%	A/Hubei/1/2010(H5N1), 93.31%	A/AmericanWigeon/SouthCarolina/22-000345-001/2021, 96.16%
Cleavage motif	PQKERRRKR↓GLF	PQKERRRKR↓GLF	PLREKRRKR↓GLF
Total mutations (Best reference hit, Flusurver)	122	129	120

Viral RNA was extracted from tracheal and/or oropharyngeal swabs using a KingFisher ML (Thermo Scientific) automated nucleic acid extraction system with the MagMAX Viral RNA Isolation Kit (Thermo Scientific), according to the manufacturer’s guidelines. Reverse transcription and PCR amplification were performed using the SuperScript III One-Step RT-PCR System with Platinum Taq DNA Polymerase (Invitrogen) and a custom oligo mix containing HPLC-purified Uni12 (5), Uni12-mod (6), and Uni13 (5) primers using the following thermal profiles of reverse transcription at 42°C for 50 min and 50°C for 10 min, followed by 94°C for 2 min; four cycles of 94°C for 30 s, 43°C for 30 s, and 68°C for 3 min 50 s; 30 cycles of 94°C for 30 s, 57°C for 30 s, and 68°C for 3 min 30 s, with a 10-s per-cycle extension increment; and a final extension of 68°C for 10 min. PCR products were analyzed by gel electrophoresis, purified with AMPure XP beads (SQK-NBD114.96), and quantified with a Qubit 4 Fluorometer (Thermo Scientific). Purified amplicons underwent end-repair and dA-tailing with the NEBNext Ultra II End Prep Module (E7646AA) and ligation by the NEBNext Quick Ligation Module (M0367L) with the Oxford Nanopore Native Barcoding Kit 96 V14 (SQK-NBD114.96). Barcoded samples were pooled, purified, and ligated to Oxford Nanopore sequencing adapters (FA-09075). The final library was purified, quantified, and prepared as per the manufacturer’s instructions. Sequencing libraries were loaded onto FLO-FLG114 (R10.4.1) cells and sequenced on the Oxford Nanopore MinION_Mk1C with MinKNOW_24.11.8 software and fast basecalling with Dorado_7.6.7.

We performed consensus genome assembly using a reference-based approach. Raw sequencing reads in FASTQ format were decompressed, merged, and adapter-trimmed with PoreChop_0.2.4 (7). Processed reads were aligned against a multi-segment influenza A panel with Minimap2_2.28 (-ax map-ont) (8). Sequences were piled up, and variants were called using BCFtools_1.22 (“mpileup” and “call”) (9) to generate initial consensus sequences with “vcfutils.pl vcf2fq” (10). Degenerate or ambiguous bases were manually inspected using IGV_2.16.0 (11), and necessary corrections were made in BioEdit_7.7 (12) to produce the final consensus sequence. All tools were run with default parameters unless otherwise specified. Features were automatically annotated during submission through SP-GenBank.

**Fig 1:**
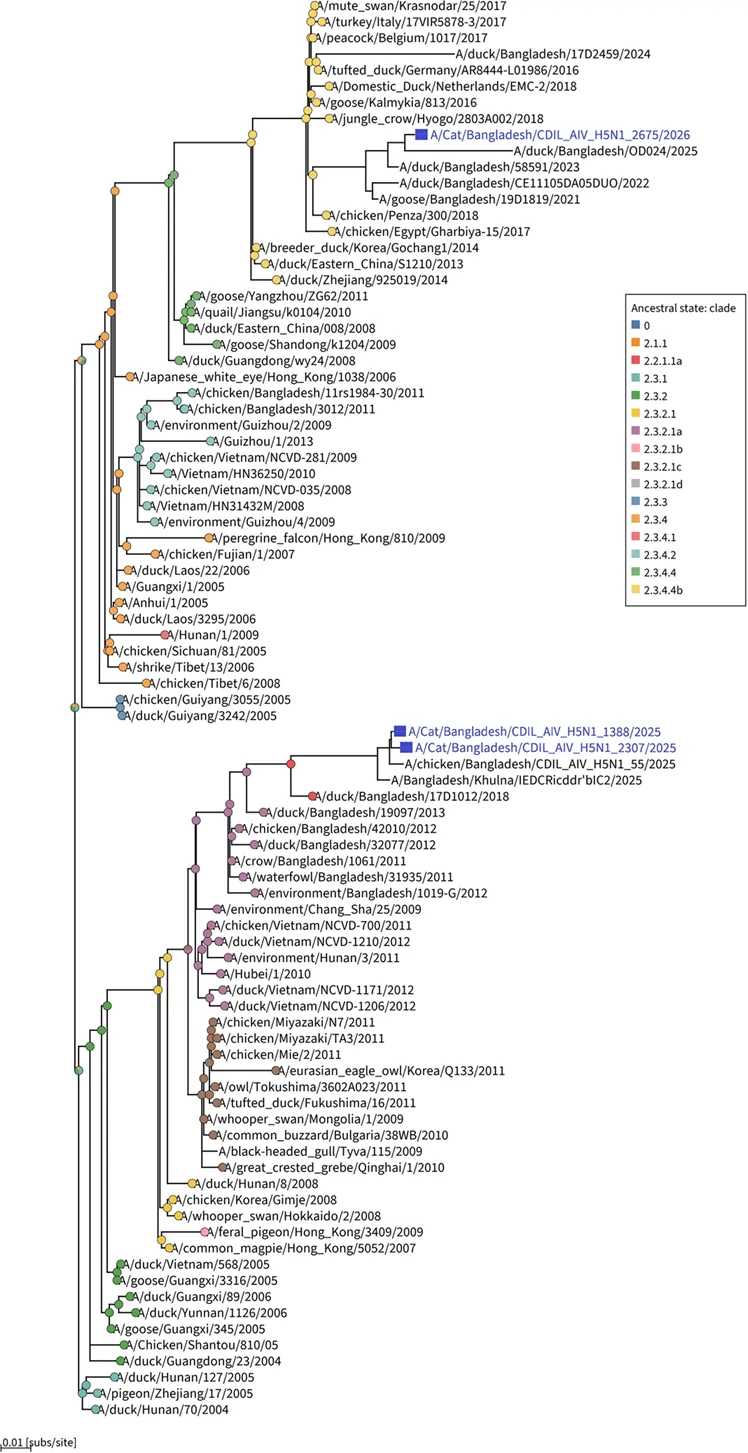
Phylogenetic tree of the H5N1 HA gene showing three cat isolates from Bangladesh. S1388 and S2307 fall in clade 2.3.2.1a; S2675 clusters in clade 2.3.4.4b among diverse avian hosts.

The coding-complete genome sequences of three Bangladeshi H5N1 strains, CDIL_AIV_H5N1_1388 (S1388), CDIL_AIV_H5N1_2307 (S2307), and CDIL_AIV_H5N1_2675 (S2675), were submitted to GenBank by CDIL (Table 1). Phylogenetic placement in Nextclade (13) assigned S1388 and S2307 to Clade 2.3.2.1a and S2675 to Clade 2.3.4.4b (Fig. 1), with polybasic cleavage sites PQKERRRKR↓GLF and PLREKRRKR↓GLF, respectively, as reported previously (14). Mutation analysis in FluSurver (15) indicated that S1388 and S2307 had high mutation counts in the surface proteins HA (25, 26) and NA (27, 30) relative to their reference sequences. S2675 is more genetically distinct, with fewer surface mutations (10 HA, 18 NA). The H5 viruses had significant substitutions associated with zoonotic risk across all segments.

Genomic analysis of the spillover events provides critical insights into mammalian adaptation mutations and enhances molecular surveillance efforts to monitor avian influenza transmission at the animal-human interface.

## Data Availability

The coding-complete genome sequence of three highly pathogenic H5N1 avian influenza A strains has been submitted to the NCBI GenBank database under SRA numbers SRR39340853, SRR39340852, and SRR39340851; accession numbers PZ602608–PZ602615, PZ602592–PZ602599, and PZ602600–PZ602607. Significant substitutions associated with zoonotic risk in the study isolates are available at https://doi.org/10.6084/m9.figshare.33993562.
